# International Federation of Gynecology and Obstetrics Global Declaration on Cervical Cancer Elimination*

**DOI:** 10.1055/s-0039-1679865

**Published:** 2019-02

**Authors:** FIGO World Congress

During the International Federation of Gynecology and Obstetrics (FIGO) World Congress, the FIGO launched the Global Declaration on Cervical Cancer Elimination.

Cervical cancer is a grave threat to the health and lives of women. Globally, 1 woman dies of cervical cancer every 2 minutes. This suffering is unacceptable, particularly since cervical cancer is largely preventable and treatable, when detected early.

Cervical cancer remains one of the most common causes of death for women, particularly the poorest and most vulnerable. Nearly 90% of the deaths from cervical cancer each year are of women living in low- and middle-income countries. Most of these women will not have had access to the key cervical cancer services that could have saved their lives, nor to the palliative care to help them manage their symptoms with dignity and respect. It should also be noted that women living with HIV are at a 4 to 5 times higher risk of having cervical cancer.

This is a story of inequality that the World Health Organization (WHO), the United Nations Population Fund (UNFPA), the FIGO and their partners aim to change dramatically. The present declaration calls on the FIGO membership to join with the WHO, with the UNFPA, and with the FIGO leadership to work towards ending the pain and anguish of women suffering from cervical cancer and eliminating this preventable tragedy, solving one of the most pressing problems in sexual and reproductive health.

The present declaration unites the FIGO membership with the WHO and the UNFPA in following the call to action by Dr Tedros, Director-General of the WHO, for “coordinated action globally to eliminate cervical cancer by making sure that all women and girls, in particular those in low-income countries, have equal access to life-saving prevention technologies and services”.

The FIGO encourages countries to reach all eligible girls and women through comprehensive cervical cancer control through a life course approach: including vaccination against human papillomavirus (HPV), screening and treatment of cervical precancerous lesions, early diagnosis, and timely treatment of invasive cancer and palliative care.

As the leading professional society in the field of gynecology and obstetrics, the FIGO has a large membership with global reach that can bring their expertise to the global call to eliminate cervical cancer in the following key areas:

- the global advocacy movement
- the need to address health workforce knowledge and skills gaps
- improvement of community engagement and health literacy
- technical assistance for global guidance
- leverage of our collaborations and partnerships

For too long, too many of the poorest and most vulnerable women have suffered from this highly preventable and curable form of cancer. With significant strides made in reducing maternal mortality, women who are saved now risk dying later from cervical cancer.

The present FIGO declaration is a commitment to collaborate on the global effort to accelerate national action towards the elimination of cervical cancer. This is to make cervical cancer a story of the past and to safeguard the human rights of all women everywhere to health and well-being, no matter where they live.

## We Declare

We, the participants of the XXII FIGO World Congress of Obstetrics and Gynecology, held in Rio de Janeiro between 14th and 19th October 2018, hereby declare that we will work collaboratively to scale up interventions with the aim of eliminating cervical cancer as a public health concern.

In line with cervical cancer elimination priority actions:

- Introduce and scale-up HPV vaccination to achieve high coverage among girls by the age of 15 years old
- Introduce and scale-up HPV screening tests for women ≥ 30 years old and ensure appropriate management
- Increase access to the diagnosis and to the treatment of cervical cancer and ensure palliative care with financial risk protection.

## We Agree To

Undertake in all countries, in our various individual and collective capacities, to support efforts to promote the following actions for impact for girls and women worldwide:

- Advocate for national cervical cancer strategies which align with the global call to elimination
- Establish capacity-building efforts to expand the knowledge and skills of our membership to contribute to national elimination programs
- Support countries in rolling out the HPV vaccine for adolescent girls, cervical cancer screening and adequate management for older women
- Ensure that the quality of care and educational programs by the FIGO promote and support regional and national elimination ambitions
- Contribute with expertise to the emerging elimination work program , particularly supporting the WHO and the UNFPA updates of guidelines and technical guidance and their dissemination
- Harness FIGO collaborations and partnerships to promote cervical cancer elimination efforts in the context of the overall well-being of women.

Rio de Janeiro, October 18, 2018

